# Supplementary material for: Circulating Neoplastic-Immune Hybrid Cells Predict Metastatic Progression in Uveal Melanoma
Source: Cancers (Basel). 2022 Sep 23;14(19):4617. doi: 10.3390/cancers14194617 (PMC9564048; doi:10.3390/cancers14194617)
Supplement: Supplementary file 1 [file cancers-14-04617-s001.zip › cancers-1899734- supplementary.pdf]

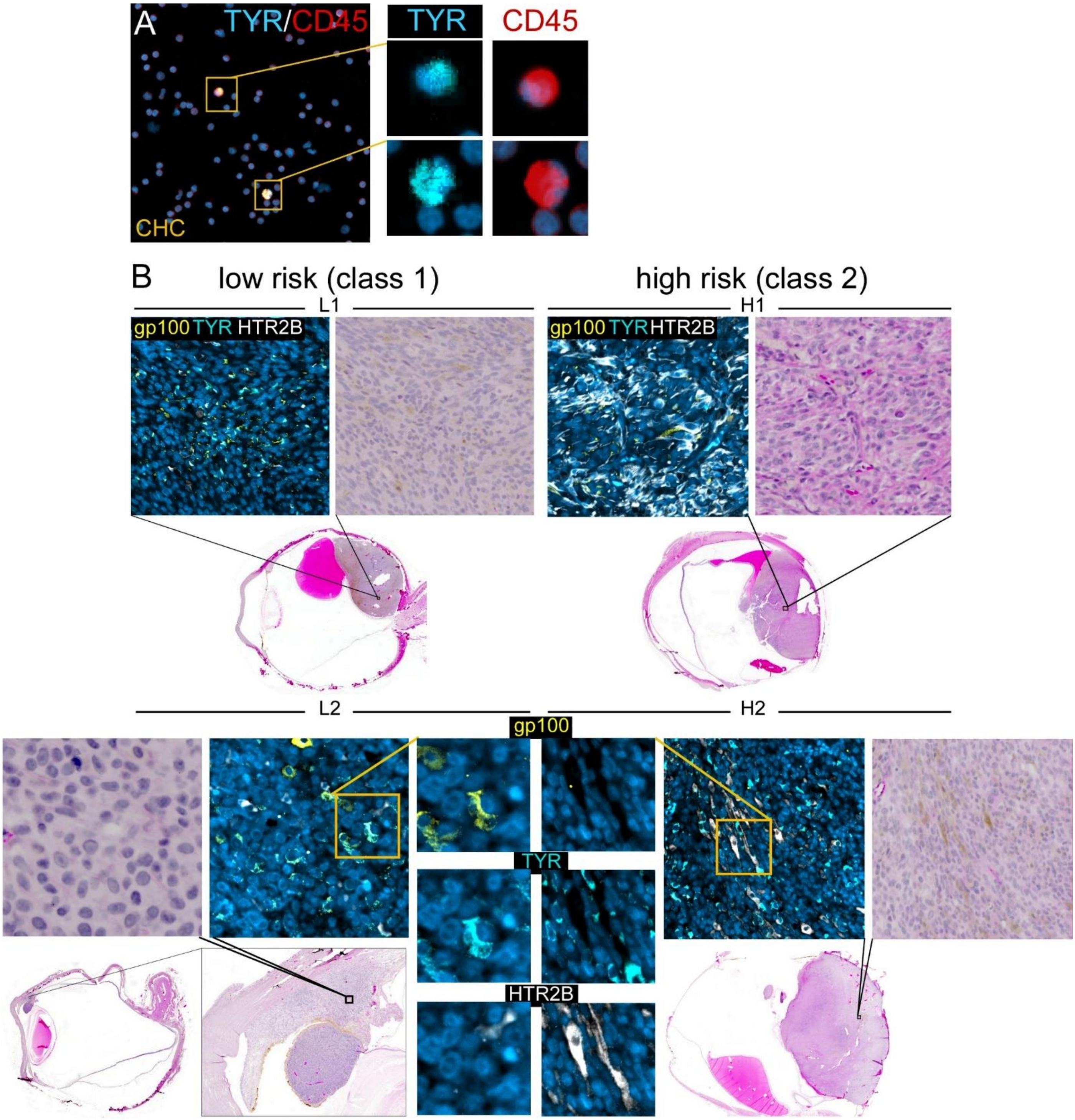

Supplemental Figure S1. Protein expression in circulating tumor cells and enucleated globes. (A) Peripheral blood mononuclear cells stained with antibodies against tyrosinase (TYR) and CD45 identify circulating hybrid cells (CHC). Higher magnification of boxed regions shown on right in single color channels. (B) Two low risk (L1, L2) and two high risk (H1, H2) tumors stained with antibodies to gp100 (yellow), TYR (blue) and HTR2B (white) merged in the image. Hematoxylin & eosin stained image of same region and of the globe is shown. For the second set of tumors, individual antibody staining shown at higher magnification of the boxed region.

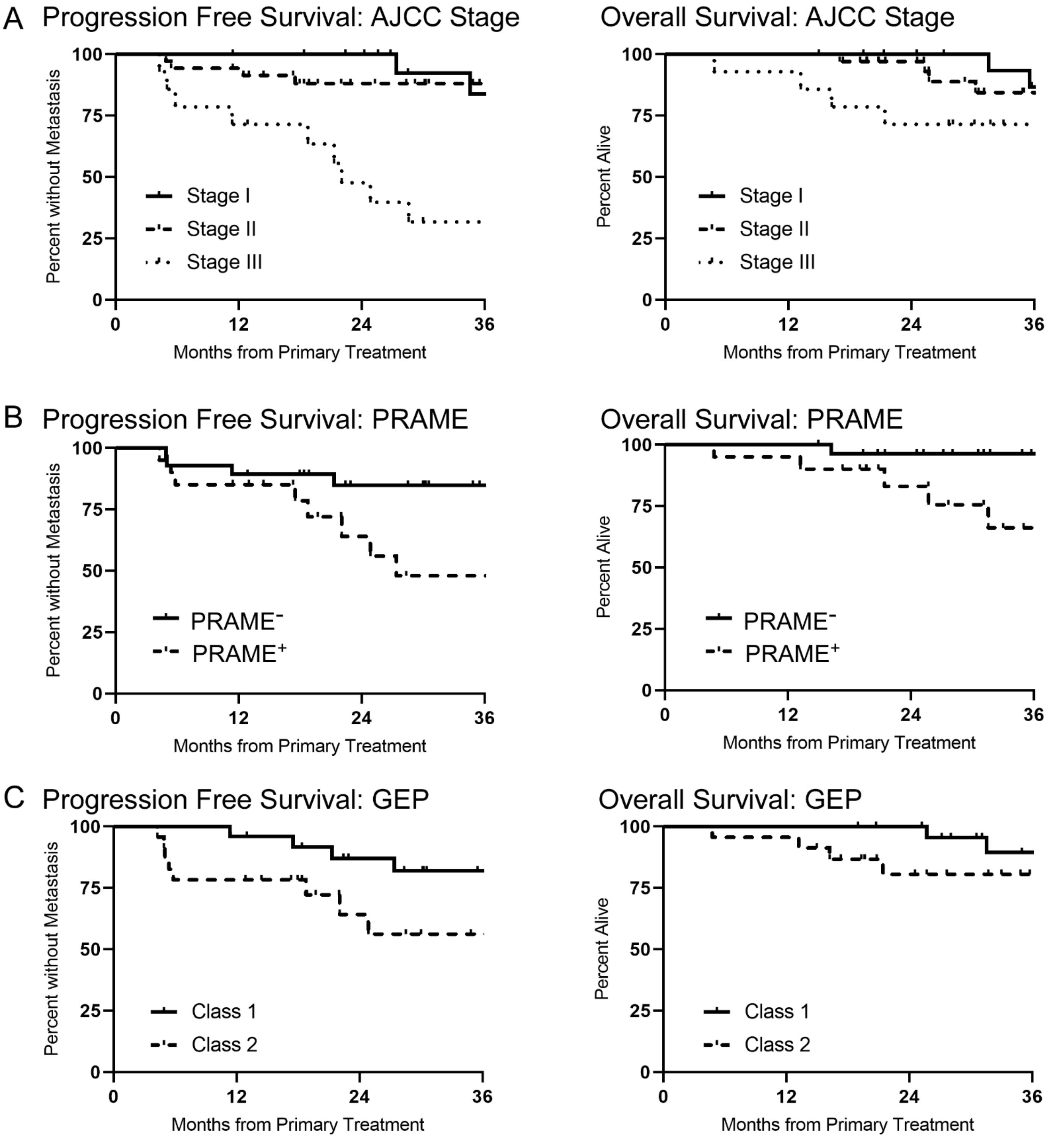

Supplemental Figure S2. Cohort characterization. All uveal melanoma subjects evaluated for progression-free survival and overall survival by AJCC stage, PRAME status and gene expression profile (GEP).

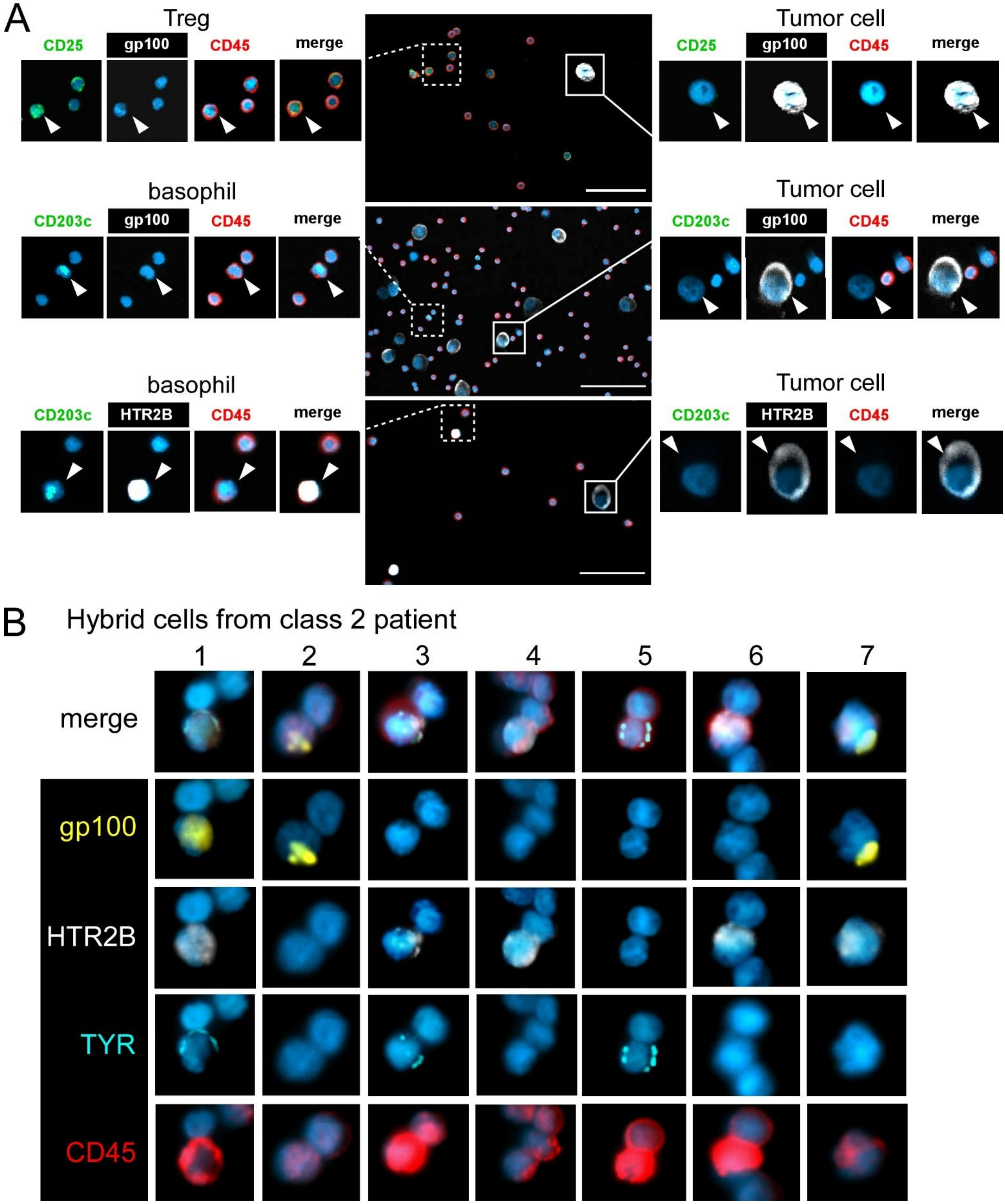

Supplemental Figure S3. Validation staining for antibodies. (A) Mixed peripheral blood mononuclear cells (PBMCs) spiked with uveal melanoma cell lines stained with antibodies to CD25 (green), CD203c (green), gp100 (white), HTR2B (white), and CD45 (red). Dashed boxes highlight immune cells that express CD25 or CD203c, solid lined boxes highlight tumor cells that express gp100 or HTR2B. (B) Seven individual hybrid cells from a class 2 uveal melanoma patient. PBMCs stained with antibodies to gp100 (yellow), HTR2B (white), TYR (blue) and CD45 (red) highlight heterogeneity among disseminated CHCs.

Supplemental Table S1. Antibodies

Target cells	Antigen	Clone	Fluor (Dye)	Manufacturer	Catalogue #	Dilution
**T-regulatory cell**						
	CD25/IL-2	SP176	(none)	abcam	ab231441	1:50
	CD25/IL-2	BC96	AF488	Invitrogen	53-0259-42	1:50
**Basophil**						
	CD203c/ENPP3	NP4D6	(none)	Novus Biologicals	NBP-1-44643	1:50
	CD203c/ENPP3	4C1H2	(none)	abcam	ab233777	1:200
	CD203c/ENPP3	Polyclonal	(none)	abcam	ab150558	1:200
	CD203c/ENPP3	NP4D6	AF488	Novus Biologicals	NBP1-44643AF488	1:100
**PBMC**						
	CD45	H130	(none)	BioLegend	304002	1:50
	CD45	H130	AF647	BioLegend	304018	1:200
	CD45	H130	AF488	Invitrogen	MACD4520	1:200
	CD45	H130	Spark YG570	BioLegend	304070	1:200
**Uveal Melanoma**						
	GP100	NKI-beteb	(none)	Novus Biologicals	NBP-2-33172	1:50
	GP100	NKI-beteb	AF647	Novus Biologicals	NBP-2-33172AF647	1:200
	GP100	NKI-beteb	(none)	abcam	ab34165	1:20
	HTR2B	Polyclonal	(none)	G-Biosciences	ITA5722-100u	1:100
	HTR2B	Polyclonal	AF647	G-Biosciences	ITA5722-100u-647	1:200
	Tyrosinase	T311	(none)	Novus Biologicals	NBP2-33160	1:25
	Tyrosinase	T311	AF594	Novus Biologicals	NBP2-33160AF594	1:50
**Fluorescent secondary antibody**						
	Mouse	Polyclonal	AF488	Jackson ImmunoResearch	715-546-150	1:500
	Rabbit	Polyclonal	AF488	Jackson ImmunoResearch	711-546-152	1:500
	Rabbit	Polyclonal	AF647	Jackson ImmunoResearch	711-605-152	1:500
	Mouse	Polyclonal	Cy3	Jackson ImmunoResearch	115-165-003	1:500
	Rabbit	Polyclonal	AF750	Invitrogen	A-21039	1:500

Supplemental Table S2. Gene list upregulated in class 2 vs. class 1

See file "UM Supplementary Table S2
